# Dysregulation of Ceramide Metabolism Is Linked to Iron Deposition and Activation of Related Pathways in the Aorta of Atherosclerotic Miniature Pigs

**DOI:** 10.3390/antiox13010004

**Published:** 2023-12-19

**Authors:** Zhaowei Cai, Liqun Deng, Yingying Fan, Yujie Ren, Yun Ling, Jue Tu, Yueqin Cai, Xiaoping Xu, Minli Chen

**Affiliations:** 1 Laboratory Animal Research Center, Academy of Chinese Medical Sciences, Zhejiang Chinese Medical University, Hangzhou 310053, China; lyun1983@163.com (Y.L.); zheyu1114@126.com (J.T.); yqcai@zcmu.edu.cn (Y.C.); xxp@zcmu.edu.cn (X.X.); 2 School of Pharmacy, Zhejiang Chinese Medical University, Hangzhou 310053, China; dengliqun@westlake.edu.cn (L.D.); fyy2514@163.com (Y.F.); ruiqing1109@126.com (Y.R.); 3 Key Laboratory of Blood-Stasis-Toxin Syndrome of Zhejiang Province, Hangzhou 310053, China

**Keywords:** atherosclerosis, lipidomics, ceramide, iron, oxidative stress, miniature pigs

## Abstract

The miniature pig is a suitable animal model for investigating human cardiovascular diseases. Nevertheless, the alterations in lipid metabolism within atherosclerotic plaques of miniature pigs, along with the underlying mechanisms, remain to be comprehensively elucidated. In this study, we aim to examine the alterations in lipid composition and associated pathways in the abdominal aorta of atherosclerotic pigs induced by a high-fat, high-cholesterol, and high-fructose (HFCF) diet using lipidomics and RNA-Seq methods. The results showed that the content and composition of aortic lipid species, particularly ceramide, hexosyl ceramide, lysophosphatidylcholine, and triglyceride, were significantly altered in HFCF-fed pigs. Meanwhile, the genes governing sphingolipid metabolism, iron ion homeostasis, apoptosis, and the inflammatory response were significantly regulated by the HFCF diet. Furthermore, C16 ceramide could promote iron deposition in RAW264.7 cells, leading to increased intracellular reactive oxygen species (ROS) production, apoptosis, and activation of the toll-like receptor 4 (TLR4)/nuclear Factor-kappa B (NF-қB) inflammatory pathway, which could be mitigated by deferoxamine. Our study demonstrated that dysregulated ceramide metabolism could increase ROS production, apoptosis, and inflammatory pathway activation in macrophages by inducing iron overload, thus playing a vital role in the pathogenesis of atherosclerosis. This discovery could potentially provide a new target for pharmacological therapy of cardiovascular diseases such as atherosclerosis.

## 1. Introduction

Cardiovascular disease remains the leading global cause of mortality, and atherosclerosis has been established as the primary underlying pathological mechanism [1]. Atherosclerosis manifests as the buildup of lipids within arterial walls, culminating in macrophage migration and smooth muscle proliferation, resulting in arterial wall hardening, thickening, and loss of elasticity [2]. Growing evidence indicates the pivotal role of aberrant lipid metabolism in the initiation and progression of atherosclerosis, potentially serving as a common thread linking various pathogenic factors, including obesity, hypertension, and diabetes [3]. Lipid-lowering therapy is indicated for patients with cardiovascular diseases, including atherosclerosis. Statins and proprotein convertase subtilisin/kexin 9 (PCSK9) inhibitors constitute the principal means to reduce serum low-density lipoprotein cholesterol (LDL-C) levels in clinical atherosclerosis treatment. Nevertheless, many patients fail to attain ideal lipid levels even with medication, potentially encountering drug intolerance and associated risks [4]. Hence, identifying new intervention targets related to abnormal lipid metabolism could offer fresh insights for preventing and treating atherosclerosis and atherosclerotic cardiovascular diseases. 

Lipidomic analysis, based on mass spectrometry, has emerged as a potent tool for investigating lipid composition in biological samples during disease states. This approach enables us to reveal the mechanisms underpinning lipid-related diseases such as atherosclerosis by quantifying changes in specific lipid categories [5]. Current clinical research on atherosclerosis mainly involves identifying lipid biomarkers from blood samples, thereby inferring their correlation with atherosclerosis due to the challenges associated with actual plaque sampling [6,7]. However, recent studies have revealed no significant correlation between arterial plaque tissue and plasma metabolites in atherosclerotic patients and mouse models [8,9]. Consequently, it is essential to directly assess changes in lipid metabolites within atherosclerotic plaques. It is now understood that miniature pigs closely resemble humans in cardiovascular anatomical structure and lipoprotein composition, offering distinct advantages for cardiovascular disease research, including atherosclerosis. Nevertheless, there is a dearth of reports regarding the alterations in lipid composition within atherosclerotic plaque in miniature pigs. Additionally, atherosclerosis represents a complex multifactorial disease, with oxidative stress, inflammation, and apoptosis playing pivotal roles in its onset and progression. However, the intricate interplay among these key factors remains largely uncharted [10]. Therefore, when studying atherosclerosis in animal models, integrating lipidomics with additional omics methods like transcriptomics can help elucidate the potential mechanisms governing specific changes in lipid metabolism and their interaction with related molecular targets. 

Herein, we conducted untargeted lipidomic profiling of atherosclerotic aortas in Wuzhishan miniature pigs subjected to either a normal chow (NC) diet or a high-fat, high-cholesterol, and high-fructose (HFCF) diet for six months. Moreover, transcriptome sequencing was employed to investigate the potential mechanisms behind HFCF diet-induced aortic ceramide accumulation, apoptosis, and inflammation in atherosclerotic pigs. Furthermore, in vitro experiments demonstrated the role of the long-chain C16 ceramide in macrophage iron overload and the activation of downstream pathways.

## 2. Materials and Methods

### 2.1. Animals

**Figure 1:**
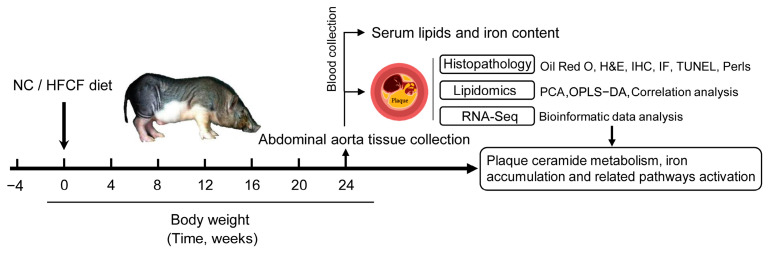
The flow diagram of the animal experiment. NC, normal chow; HFCF, high-fat, high-cholesterol, and high-fructose diet; H&E, hematoxylin and eosin; IHC, immunohistochemistry; IF, immunofluorescence; TUNEL, terminal deoxynucleotidyl transferase-mediated dUTP nick end labeling; PCA, principal component analysis; and OPLS-DA, orthogonal partial least-squares-discriminant analysis.

Ten male Wuzhishan miniature pigs aged 6–7 months were acquired from the Institute of Animal Sciences, Hainan Academy of Agricultural Sciences (Haikou, China), in this study. Each pig was individually housed and had a 12 h light and dark cycle. Before the experiment, the pigs were fed a regular diet and had free access to water. After 4 weeks of acclimatization, the animals were divided into two groups and fed either an NC (3.2 kcal·g^−1^, 22% of kcal from protein, 8% of kcal from fat, and 70% of kcal from carbohydrate) or an HFCF (4.5 kcal·g^−1^, 12% of kcal from protein, 46% from fat, and 42% from carbohydrate) diet for 6 months. Each group consisted of five animals. The HFCF diet consisted of 15% shortening oil, 10% egg yolk power, 1.5% cholesterol, 10% fructose, and 63.5% normal chow diet. The pigs in each group were fed twice daily with a total daily amount equivalent to 2.5% of their body weight. The Laboratory Animal Management and Use Committee of the Zhejiang Chinese Medical University (Hangzhou, China, permit no. ZSLL-2016-0013) authorized all procedures for this study. A diagram of the animal experiment is shown in Figure 1.

The body weight of the pigs was recorded monthly. After 6 months of feeding, the pigs were killed by exsanguination under anesthesia. Blood samples were collected and centrifuged at 3000× *g* for 15 min. The serum was separated and stored at −80 °C for further analysis. The entire aorta, from the aortic arch to the iliac artery, was removed and fixed in 10% buffered formalin for 24 h. The samples were then stained with Sudan IV dye for 15 min, rinsed, and then photographed. Image J software (NIH, version 1.6.0) was used to analyze lipid deposition in the whole aorta. 

### 2.2. Biochemical Parameter Measurements

Biochemical markers associated with lipid metabolism, including triglycerides (TG), total cholesterol (TC), high-density lipoprotein cholesterol (HDL-C), and LDL-C, were measured with an automatic biochemistry analyzer (Hitachi 7020; Hitachi, Tokyo, Japan). Iron content in the abdominal aorta tissues was determined with an iron detection kit (TC1015, Leagene, Beijing, China). Briefly, 20 mg of abdominal aorta was homogenized, and the protein concentration was measured using a bicinchoninic acid (BCA) protein assay kit. The absorbance of the collected samples was measured at the wavelength of 562 nm using a microplate reader, and the concentration of iron was calculated accordingly [11].

### 2.3. Histological Analysis

The abdominal aorta was fixed in 10% formalin, embedded in paraffin, sectioned at 4 μm thickness, and stained with hematoxylin and eosin (H&E). The intima to media thickness ratio was measured with Image J software (NIH, version 1.6.0). The sections were examined via a light microscope (Nikon Eclipse 80i; Nikon, Tokyo, Japan). Apoptosis in abdominal aorta tissues was assessed using a TUNEL BrightGreen Apoptosis Detection Kit (A112-01, Vazyme, Nanjing, China) according to the manufacturer’s instructions. The apoptotic cells were labeled with green immunofluorescence, while the cell nuclei were stained with DAPI. In brief, following rehydration with a xylene and ethanol series, the slides were treated with 20 μg/mL of proteinase K and incubated at 37 °C for 20 min. They were then rinsed with 1× phosphate buffer solution (PBS) for 5 min, three times. Subsequently, the slides were exposed to 50 μL of TUNEL detection solution and incubated at 37 °C for 60 min, followed by three washes with 1 × PBS. Finally, they were stained with an anti-fluorescence quenching solution. The rate of apoptotic cells was calculated as the ratio of TUNEL-positive nuclei to DAPI-stained nuclei. Movat staining was employed to examine foam cells and collagen fibers in the plaque. Image-Pro Plus 6.0 Software was used for area quantification.

### 2.4. Lipid Sample Preparation and Lipidomic Profiling

Lipids were extracted from abdominal aorta tissues using the methyl tert-butyl ether (MTBE) method as previously described [12]. Briefly, 30 mg of tissue was homogenized in 200 μL of ice-cold water and 240 μL of methanol, followed by 800 μL of MTBE. The samples were ultrasonically treated for 20 min at 4 °C, then allowed to rest for 30 min at room temperature. Lipids were extracted by centrifuging the solution at 10 °C for 15 min (14,000× *g*) to extract lipids. The upper organic layer was collected, and the solvent was evaporated under nitrogen and stored at −80 °C until analysis.

LC-MS/MS analysis was performed using a Q Exactive plus mass spectrometer (Thermo Scientific, Waltham, MA, USA) coupled to a UHPLC Nexera LC-30A system (Shimadzu, Tokyo, Japan). Briefly, lipids were separated using a Waters Acquity UHPLC system with a CSH C18 column (2.1 × 100 nm, 1.7 μm). MS analysis was performed using electrospray ionization (ESI) positive and negative ions. Positive and negative ion full-scan spectra were collected within mass-to-charge ratio (*m*/*z*) ranges of 200–1800 and 250–1800, respectively. Following each full scan, 10 fragment patterns (MS2 scan, HCD) were collected to determine the mass-to-charge ratio of lipid molecules. The initial lipidomic data were processed using LipidSearch software version 4.1 (Thermo Scientific^TM^, Waltham, MA, USA) for peak recognition, lipid identification (secondary identification), peak extraction, peak alignment, and quantification. Among the extracted ion features, only variables with at least 50% nonzero values were retained. Unsupervised multivariate analyses and fatty acyl chain composition analyses were carried out on identified lipids. Samples from each group were pooled equally to create a pooled quality control (QC). The experimental process included QC samples to evaluate system stability and data reliability.

### 2.5. Unsupervised Multivariate Data Analyses

Multivariate statistical analysis was conducted using SIMCA-P 16.0 software (Umetrics, Umea, Sweden). Following the Pareto scaling, principal component analysis (PCA) and orthogonal partial least-squares-discriminant analysis (OPLS-DA) were performed. By combining the VIP value obtained from the OPLS-DA model with a two-tailed Student’s *t*-test, significant differential metabolites were determined. Moreover, metabolites with VIP values greater than 1.0 and *p* values less than 0.05 were significant and considered differentially expressed. Hierarchical cluster analysis was performed with R software (version 3.5.1).

### 2.6. cDNA Library Construction and RNA-Seq Analysis

Pigs from each group were used for RNA sequencing. TRIzol reagent (Invitrogen, Carlsbad, CA, USA) was used to isolate total RNA from abdominal aorta tissues. Quantity and purity of RNA were assessed using the Bioanalyzer 2100 (Agilent, Santa Clara, CA, USA) and Animal Tissue RNA Purification Kit (LC Sciences, Houston, TX, USA), respectively. 

RNA samples of high quality were used for cDNA library construction. More detailed information on the cDNA library construction, paired-end sequencing, quality control, reads mapping, and the fragments per kilobase of exon per million reads mapped (FPKM) calculations can be found in our previous studies [13,14]. Using the Illumina paired-end RNA-Seq approach, we sequenced the transcriptome, generating a total of a million 2 × 150 bp paired-end reads. We aligned reads of all samples to the sus scrofa reference genome (Ssscrofa 10.2) using the HISAT2 (https://daehwankimlab.github.io/hisat2/, accessed on 27 March 2021) package. The mapped reads of each sample were assembled using StringTie (http://ccb.jhu.edu/software/stringtie/, accessed on 27 March 2021). Differentially expressed genes (DEGs) were selected based on a fold change > 2 or <0.5 with statistical significance determined with a *p*-value < 0.05 using R language. The raw data were deposited into the Gene Expression Omnibus (GEO) database under accession number GSE245530.

### 2.7. Bioinformatic Analysis

Gene Ontology (GO) annotation and Kyoto Encyclopedia of Genes and Genomes (KEGG) pathway enrichment analysis were applied to analyze the main function of DEGs according to the NCBI Gene Ontology database. An adjusted *p*-value < 0.05 was set as the threshold for significantly enriched GO terms and KEGG pathways. We performed gene set enrichment analysis using the software Gene Set Enrichment Analysis (GSEA, version 4.3.2) to identify whether a set of genes in Reactome shows significant differences in the two groups. The normalized enrichment score (NES) > 1, nominal (NOM) *p*-value < 0.05, and false discovery rate (FDR) *q*-value < 0.25 were used to highlight significant pathways of enrichment.

### 2.8. Cell Culture

RAW264.7 cells were purchased from the Cell Resource Center of the Chinese Academy of Medicinal Sciences (Beijing, China). RAW264.7 cells were cultured at 37 °C in a 5% CO_2_ atmosphere. The DMEM was supplemented with 10% fetal bovine serum and contained 100 U/mL of penicillin and 100 μg/mL of streptomycin. RAW264.7 cells were seeded in triplicates in 96-well plates at a certain density (2 × 10^5^ cells/well). Then, the cells were treated with different concentrations of C16 ceramide (860516P, Avanti Polar Lipids) (1, 10, 100, and 200 μM) for 24 h. After 24 h of incubation, 50 μL of MTT reagent was added to each well and cultured in an incubator for 4 h in the dark. The supernatant was removed, and the optical density of the formazan solution was measured by using a microplate reader at 570 nm. 

To investigate the impact of C16 ceramide on cellular iron homeostasis and function, RAW264.7 cells underwent different treatments, including PBS, 100 μM C16 ceramide, and 50 μM deferoxamine (DFO) (HY-B0988, MCE). For the measurement of reactive oxygen species (ROS), cells from the various groups were first washed twice with PBS and then incubated with the 2′,7′-dichlorodihydro-fluorescein diacetate (DCFH-DA) probe (10 μmol/L) (S0033S, Beyotime, Shanghai, China) for 20 min at 37 °C. The fluorescence intensity was subsequently determined at an excitation wavelength of 488 nm and an emission wavelength of 525 nm using a fluorescence microscope. For flow cytometry analysis, cells were harvested and washed twice in ice-cold PBS after 24 h of treatment. Then, the cells were resuspended in 100 μL of 1 × Binding Buffer provided in the kit. To each tube, 5 μL of Annexin V-PE and 5 μL of 7-AAD were added, mixed gently, and incubated for 15 min in the dark at room temperature. Finally, 400 μL of 1 × Binding Buffer was added to the samples, and they were promptly subjected to flow cytometry analysis (Beckman Coulter, Brea, CA, USA).

### 2.9. Quantitative Real-Time PCR Analysis

Real-time PCR analysis was performed as previously described [14]. Briefly, total RNA was isolated from abdominal aorta tissues using TRIzol reagent (Invitrogen). One microgram of total RNA was reverse-transcribed using random primers and MMLV reverse transcriptase (Promega, Madison, WI, USA). Quantitative Real-time PCR (qRT-PCR) was carried out using the StepOnePlus Real-Time PCR Detection System (Applied Biosystems, Foster City, CA, USA). Relative expression levels of genes were analyzed using the 2^−∆∆CT^ method. The primers used are listed in Supplementary Materials, S1: Table S1.

### 2.10. Western Blot Analysis

Western blotting was conducted as previously described [13,14]. Briefly, total protein was isolated from frozen abdominal aorta tissues. Then, the cells were lysed using RIPA buffer and further quantified using a BCA protein assay Kit (KGP2100, Keygen, China). Next, the proteins were subjected to SDS-PAGE and electrotransferred onto PVDF membranes (Bio-Rad, Hercules, CA, USA). Then, the membranes were incubated with primary antibodies overnight at 4 °C, followed by the corresponding secondary antibodies (Li-COR Biosciences, Lincoln, NE, USA) for 1 h at room temperature. The primary antibodies used were as follows: β-actin (4970S, Cell Signaling Technology, CST, Boston, MA, USA), Caspase-3 (9662S, CST, USA), Caspase-8 (4790T, CST, Boston, USA), Caspase-9 (9508S, CST, Boston, MA, USA), NF-қB p65 (8242S, CST, Boston, MA, USA), Phospho-NF-қB p65 (Ser536) (3033T, CST, Boston, MA, USA), TLR2 (13744S, CST, USA), TLR4 (A00017S441, Boster, Wuhan, China), p38 MAPK (9212S, CST, USA), Phospho-p38 MAPK (Thr180/Tyr182) (4613S, CST, Boston, MA, USA), BCL-2 (ab32124, Abcam, Cambridge, MA, USA), Bax (ab32503, Abcam, Cambridge, MA, USA), and TFRC (ab269513, Abcam, Cambridge, MA, USA). The target protein bands were visualized using the Odyssey Infrared Imaging System (Li-COR Biosciences, USA). 

### 2.11. Immunohistochemistry and Immunofluorescence Staining

As previously described [13], the expression of NF-қB p65 (6956T, CST, Boston, MA, USA), α-SMA (ab5694, Abcam, Cambridge, MA, USA), CD68 (ab201340, Abcam, Cambridge, MA, USA), TFRC (ab269513, Abcam, Cambridge, MA, USA), and FTL (ab269513, Abcam, Cambridge, MA, USA) in the abdominal aorta tissues were assessed via immunostaining. Briefly, sections were blocked with a 3% hydrogen peroxide (H_2_O_2_) to block the endogenous peroxidase. Then, the sections were incubated overnight with primary anti-NF-қB p65, anti-α-SMA, anti-TFRC, and anti-FTL antibodies at 4 °C. Next, washing was performed and the secondary antibody was incubated for 1 h at room temperature. After washing, sections were counterstained with hematoxylin and visualized with a diaminobenzidine (DAB) kit (Vector Laboratories, Burlingame, CA, USA). For immunofluorescence staining, the sections were incubated with primary anti-CD68 overnight at 4 °C. Afterwards, the sections were incubated with the appropriate fluorescently labeled secondary antibodies for 1 h at room temperature. After washing, DAPI (Roche) was used to stain the nuclei for 8 min. Images were taken with a DS-Fil digital camera mounted on a Nikon Eclipse 80i fluorescence microscope (Nikon).

### 2.12. Statistical Analysis

GraphPad Prism 8.0 (Graphpad Software Inc., San Diego, CA, USA) was used for statistical calculations and data plotting. Statistical differences between two groups of unpaired design were determined using the Student’s *t*-test. The comparison among multiple groups was conducted using one-way analysis of variance (ANOVA) and followed by Tukey’s test. The results are presented as mean ± standard error. A *p*-value of less than 0.05 was considered statistically significant.

## 3. Results

### 3.1. Phenotype of Atherosclerotic Pigs Induced by High-Fat, High-Cholesterol, and High-Fructose Diet

**Figure 2:**
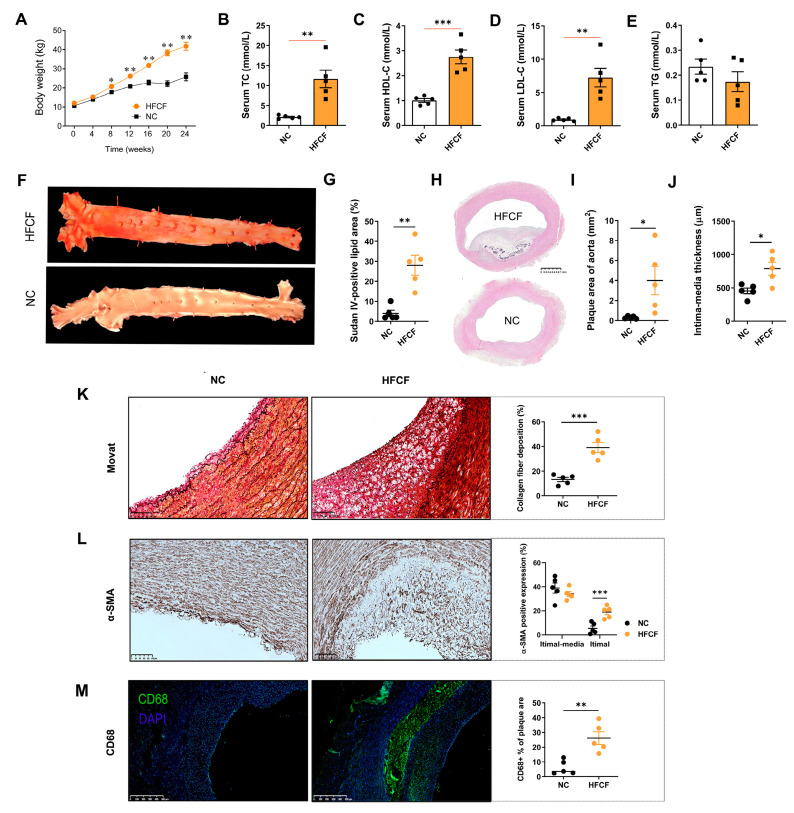
Phenotype of atherosclerotic pigs induced by a high-fat, high-cholesterol, and high-fructose diet. (**A**) Body weight of pigs during the dietary intervention time; (**B**) serum total cholesterol; (**C**) serum high-density lipoprotein cholesterol; (**D**) serum low-density lipoprotein cholesterol; (**E**) serum triglyceride; (**F**) representative photographs of Sudan-IV-stained aorta; (**G**) quantification of the Sudan-IV-positive lesion area in en face aorta; (**H**) H&E staining of the abdominal aorta; scale bars, 1 mm; (**I**) mean plaque size of abdominal aortas; (**J**) intima-media thickness of abdominal aortas; (**K**) representative photomicrographs and quantification of Movat staining in sections of the abdominal aorta; (**L**) representative photomicrographs and quantification of α-SMA immunohistochemistry in sections of the abdominal aorta; and (**M**) representative photomicrographs and quantification of CD68 staining in sections of the abdominal aorta. Green, CD68; blue, DAPI. Original magnification, ×50. NC, normal chow; HFCF, high-fat, high-cholesterol, and high-fructose diet. All data were assessed using Student’s *t*-test and are presented as mean ± SEM, *n* = 5 per group. * *p* < 0.05, ** *p* < 0.01, *** *p* < 0.001.

Pigs on the HFCF diet exhibited significant weight gain (29.9 ± 5.1 kg vs. 14.2 ± 4.8 kg) and higher serum cholesterol levels, including TC (*p* < 0.01) and LDL-C (*p* < 0.01), compared with the pigs in the normal chow group (Figure 2A,B,D). No significant differences in TG concentrations were observed between the HFCF and NC groups. Sudan IV staining revealed a substantial increase in atherosclerotic lesion areas in HFCF-fed pigs (Figure 2F,G). Histological examination of H&E sections showed that the intimal area and intima-media thickness of aortas in the HFCF group were 13.0- and 1.5-fold higher than those in the NC group (*p* < 0.05) (Figure 2H–J). Distinctive features of atherosclerosis, including foam cell infiltration, necrotic core formation, and smooth muscle cell proliferation, were observed in arterial tissues of the HFCF group (Figure 2H). Sections were stained with Movat pentachrome to assess intimal and medial areas and plaque classification, while α-SMA staining confirmed collagen deposition and smooth muscle cell migration (Figure 2K,L). Moreover, increased CD68-positive staining was observed in the aorta of the HFCF group (*p* < 0.01) (Figure 2M).

### 3.2. Alteration in Aortic Lipidomics in Atherosclerotic Pigs Induced by High-Fat, High-Cholesterol, and High-Fructose Diet

**Figure 3:**
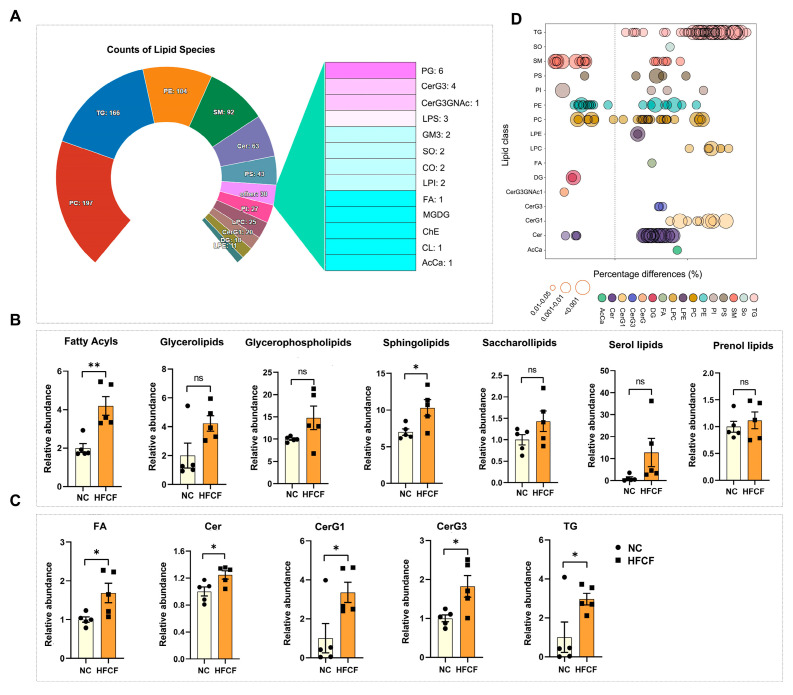
Changes in the overall lipid composition and distribution in the aorta of atherosclerotic pigs fed a high-fat, high-cholesterol, and high-fructose diet. (**A**) Distribution of lipid classes considered for subsequent analysis in all of the samples detected via LC-MS/MS; (**B**) the intensity fold change in fatty acyls, glycerolipids, glycerophospholipids, sphingolipids, saccharolipids, and prenol lipids; (**C**) the intensity fold change in AcCa, FA, Cer, CerG1, CerG3, and TG; only lipids with *p* < 0.05 are displayed; and (**D**) bubble plot of altered lipid species in the aorta of atherosclerotic pigs fed a HFCF diet. Each dot represents a lipid species, and the dot size indicates significance. NC, normal chow; HFCF, high-fat, high-cholesterol, and high-fructose diet; ns, no significant. All data were assessed using Student’s *t*-test and are presented as mean ± SEM, *n* = 5 per group. * *p* < 0.05, ** *p* < 0.01.

To explore changes in the overall lipid composition and distribution in the aorta of atherosclerotic pigs fed the HFCF diet, we conducted mass spectrometry-based lipidomic analysis on abdominal aorta tissues from both the HFCF and the NC groups. The detected lipid classes and their abbreviations are listed in the Supplementary Materials, S1: Table S2 and S2: Table S3. We identified more than 796 distinct lipid species in abdominal aorta tissue, comprising 197 phosphatidylcholines (PCs), 166 triacylglycerols (TGs), 104 phosphatidylethanolamines (PEs), 92 sphingomyelins (SMs), 63 ceramides (Cers), 25 lysophosphatidylcholines (LPCs), 20 hexosyl ceramides (CerG1), and other lipid classes (Figure 3A). Using OPLS-DA, we identified two separate groups (HFCF vs. NC; positive ion mode: R^2^X = 0.834, R^2^Y = 0.999, and Q2 = 0.897; negative ion mode: R^2^X = 0.852, R^2^Y = 0.998, and Q2 = 0.900; Supplementary Materials, S1: Figure S1). Approximately 23% of the identified lipid species exhibited significant changes, with 139 lipid species increased and 42 decreased. Grouping the quantification results based on lipid categories, we observed a significant increase in the total abundance of fatty acyls and sphingolipids. In contrast, the abundance of glycerolipids, glycerophospholipids, saccharolipids, sterol lipids, and prenol lipids remained unaltered. A more detailed analysis of lipid subclasses showed increased levels of acylcarnitine (AcCa), fatty acids (FA), Cer, CerG1, CerG3, and TG in the HFCF group (Figure 3B,C). Additionally, LPC and lysophosphatidylethanolamine (LPE) levels in glycerophospholipids in the HFCF group tended to be higher than in the NC group (*p* = 0.069 and 0.079). To further investigate individual lipid species regulated by the HFCF diet, significantly altered lipid species were visualized in a bubble plot (Figure 3D). 

**Figure 4:**
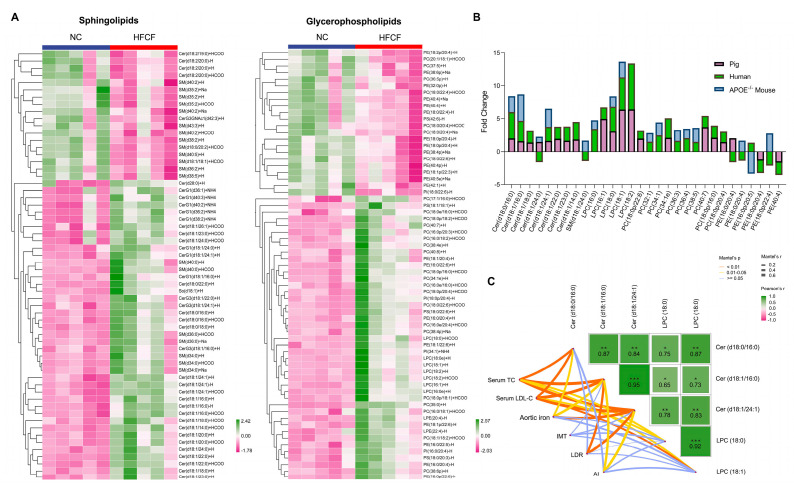
Comparison of significantly altered lipids in the atherosclerotic aorta of pigs and other species. (**A**) Heatmap showing the differentially expressed sphingolipid and glycerophospholipid subclasses; (**B**) Log2 (fold change) values of significantly altered lipids in the atherosclerotic aorta of humans, pigs, and APOE^−/−^ mice; and (**C**) pairwise comparisons of significantly altered lipids are shown with a color gradient denoting Spearman’s correlation coefficient. NC, normal chow; HFCF, high-fat, high-cholesterol, and high-fructose diet; IMT, intima-media thickness; LDR, lipid deposition rate; and AI, atherosclerotic index. All data were assessed using Student’s *t*-test and are presented as mean ± SEM, *n* = 5 per group. * *p* < 0.05, ** *p* < 0.01, *** *p* < 0.001.

We employed hierarchical clustering heatmaps to illustrate changes in lipid subclasses of sphingolipids and glycerophospholipids (Figure 4A). In comparison with the NC group, we observed a significant increase in twenty-six ceramides, including Cer d18:0/16:0, Cer d18:0/18:0, Cer d18:0/22:0, Cer d18:1/14:0, Cer d18:1/18:0, Cer d18:1/20:0, Cer d18:1/22:0, Cer d18:1/24:0, Cer d18:1/24:1, and Cer d18:2/20:0, as well as twenty sphingomyelins, including SM d34:0, SM d36:0, and SM d40:0, in the abdominal aorta of HFCF-fed pigs (Figure 4A). Seven LPC lipid species, such as LPC (16:0e), LPC (16:1), LPC (18:0), LPC (18:0e), LPC (18:1), and LPC (18:2), and two LPE lipid species, including LPE (20:4) and LPE (22:4), were also significantly elevated in the abdominal aorta of the HFCF group (Figure 4A).

We analyzed lipid changes in plaques from atherosclerotic pigs, APOE^−/−^ mice, and human patients. We found five common lipid species, including Cer (d18:0/16:0), Cer (d18:1/16:0), Cer (d18:1/24:1), LPC (18:0), and LPC (18:1), which were significantly increased in atherosclerotic plaques (Figure 4B). We also analyzed the correlation between these common lipids and atherosclerotic parameters and found positive correlations with serum cholesterol content, intima–media thickness, plaque area, and, intriguingly, plaque iron content, especially with Cer (d18:1/16:0) (C16 Cer) (Figure 4C), suggesting that ceramides might contribute to atherosclerosis by influencing iron deposition. 

Maintaining cell membrane homeostasis is crucial for cardiovascular health, with the carbon chain length and degree of saturation of fatty acyls in the membrane lipids influencing membrane biophysical properties [15]. We further analyzed the fatty acyl chain profile associated with sphingolipids. Our data indicated that the HFCF diet led to a substantial alteration in the fatty acyl chain profile of ceramides, CerG1, and CerG3, with increased levels of long-chain fatty acyls with 34C and polyunsaturated fatty acyls with low double bond numbers (1~2 double bonds) in the abdominal aorta tissue of pigs (Supplementary Materials, S1: Figure S2). Regarding the effects of the HFCF diet on sphingolipid composition, we observed an increase in short-chain fatty acyls (16C) and medium-length (24C) fatty acyls in the abdominal aorta tissue. Most of the fatty acyl chain profiles of sphingomyelins remained unaltered due to HFCF diet feeding (Supplementary Materials, S1: Figure S2). These findings suggest that the fatty acyl chains associated with sphingolipids undergo dynamic changes after HFCF diet feeding.

### 3.3. Significantly Increased Ceramide Metabolism, Inflammation, and Apoptosis-Related Genes Were Observed in the Aorta of Atherosclerotic Pigs Induced by High-Fat, High-Cholesterol, and High-Fructose Diet

We applied RNA sequencing (RNA-Seq) to map the transcriptional alterations in the abdominal aorta tissue. We identified 3396 DEGs, including 2652 upregulated and 744 downregulated genes (Supplementary Materials, S2: Table S4). 

**Figure 5:**
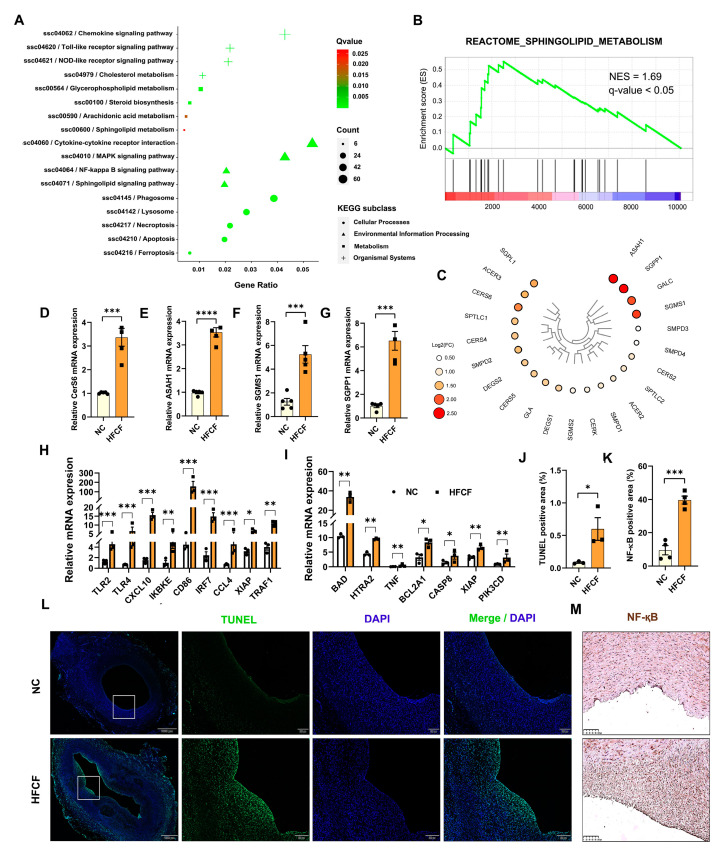
Alterations in the aortic transcriptional profiles of atherosclerotic pigs fed a high-fat, high-cholesterol, and high-fructose diet. (**A**) KEGG pathway analysis showing the enrichment of functional categories. The size of shapes indicates gene counts. The dot color indicates significance, and corresponding significance values are displayed as log10 (*p*-value). (**B**) Gene set enrichment analysis showing significant enrichment in sphingolipid metabolism. (**C**) Heatmap showing the ceramide-generation-related genes. The greater the log2 (fold change), the higher the significance. (**D**–**G**) qRT-PCR analysis of CERS6, ASAH1, SGMS1, and SGPP1 (*n* = 5). (**H**) relative expression levels of selected genes involved in the NF-қB signaling pathway from the RNA-Seq dataset; (**I**) relative expression levels of selected genes involved in apoptosis from the RNA-Seq dataset. (**J**) The quantification of TUNEL-positive cells (*n* = 3). (**K**) The quantification of NF-қB-positive cells (*n* = 4). (**L**) Representative photographs of TUNEL-stained aorta of the NC and HFCF groups of pigs. Green, TUNEL; blue, DAPI. Scale bars, 200 and 1000 μm. (**M**) Representative photomicrographs of NF-қB staining in sections of the abdominal aorta in the NC and HFCF groups of pigs. Scale bars, 100 μm. NC, normal chow; HFCF, high-fat, high-cholesterol, and high-fructose diet. All data were assessed using Student’s *t*-test and are presented as mean ± SEM. * *p* < 0.05, ** *p* < 0.01, *** *p* < 0.001, **** *p* < 0.0001.

For a comprehensive understanding of the functions of these DEGs, we conducted GO and KEGG pathway analyses. KEGG pathway analysis revealed significant enrichment in several immune and inflammatory response pathways, including the chemokine signaling pathway, mitogen-activated protein kinase (MAPK) signaling pathway, toll-like receptor signaling pathway, NF-kappa B signaling pathway, and NOD-like receptor signaling pathway. Interestingly, multiple lipid metabolism pathways, such as steroid biosynthesis, sphingolipid signaling pathway, sphingolipid metabolism, glycerophospholipid metabolism, and arachidonic acid metabolism, were also significantly enriched. Moreover, other pathways, including apoptosis and ferroptosis, exhibited significant enrichment (Figure 5A, Supplementary Materials, S2: Table S5). GO annotation revealed significantly enriched biological processes in the aorta of the HFCF group of pigs, encompassing inflammatory response, positive regulation of MAP kinase activity, positive regulation of the MAPK cascade, toll-like receptor 4 signaling pathway, response to lipid, regulation of the apoptotic process, reactive oxygen species metabolic processes, iron ion homeostasis, and cellular iron ion homeostasis (Supplementary Materials, S2: Table S6). We further performed GSEA to identify the biological processes in atherosclerotic pigs. The results revealed significant changes in sphingolipid metabolism, iron uptake and transport, toll-like receptor cascades, and apoptosis (Figure 5B, Supplementary Materials, S1: Figure S3). Taken together, these results indicate that HFCF diet feeding can lead to alterations in multiple signaling pathways in the abdominal aorta tissue of pigs.

We generated heat maps to depict the expression of genes enriched in sphingolipid metabolism. Among these genes, ceramide synthase 6 (CERS6), sphingomyelin synthase 1 (SGMS1), neutral ceramidase 1 (ASAH1), sphingosine-1-phosphate phosphatase 1 (SGPP1), sphingosine-1-phosphate lyase 1 (SGPL1), and galactosylceramidase (GALC) displayed high expression levels in the abdominal aorta of atherosclerotic pigs (Figure 5C), suggesting that HFCF diet feeding promotes ceramide accumulation in the abdominal aorta of pigs. Ceramide synthesis in the abdominal aorta tissue was validated via qRT-PCR analysis, which confirmed significantly increased mRNA levels of CERS6, SGPP1, ASAH1, and SGMS1 in the HFCF group (*p* < 0.001) (Figure 5D–G). 

The above-mentioned results consistently revealed significant enrichment of apoptosis and the NF-kappa B signaling pathway. Several key genes associated with the NF-kappa B signaling pathway, including toll-like receptor 2 (TLR2), TLR4, inhibitor of nuclear factor kappa B kinase subunit epsilon (IKBKE), CC cytokine ligand-4 (CCL4), C-X-C motif chemokine ligand 10 (CXCL10), interferon regulatory factor 7 (IRF7), and tumor necrosis factor-associated factor 1 (TRAF1), exhibited increased expression in the abdominal aorta tissue of the HFCF group (Figure 5H). Genes related to apoptosis, such as cathepsin D (CTSD), Bcl2-associated agonist of cell death (BAD), tumor necrosis factor (TNF), and Caspase-8 (CASP8), were also upregulated in the abdominal aorta tissue of the HFCF group (Figure 5I). TUNEL staining and NF-қB immunohistochemical staining confirmed increased apoptosis (*p* < 0.05) and NF-қB protein expression (*p* < 0.001) in the abdominal aorta tissue of the HFCF group, consistent with the RNA-Seq results (Figure 5J–M).

### 3.4. Significantly Increased Iron Metabolism-Related Genes Were Observed in the Aorta of Atherosclerotic Pigs Induced by High-Fat, High-Cholesterol, and High-Fructose Diet

**Figure 6:**
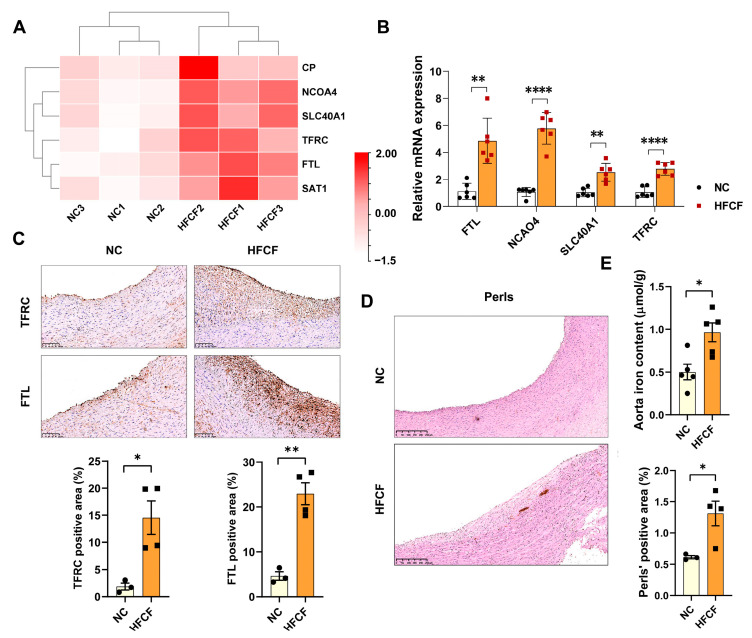
Iron deposition in the aorta of atherosclerotic pigs induced by a high-fat, high-cholesterol, and high-fructose diet. (**A**) Heatmap showing the differentially expressed genes related to iron accumulation; (**B**) qRT-PCR analysis of TFRC, FTL, SLC40A1, and NCOA4 (*n* = 5); (**C**) representative photomicrographs and quantifications of TFRC and FTL staining in sections of the abdominal aorta in the NC and HFCF groups of pigs (*n* = 3); scale bars, 100 μm; (**D**) representative photomicrographs and quantifications of DAB-enhanced Perls’ iron staining of the aorta in the NC and HFCF groups of pigs (*n* = 3–4); scale bars, 250 μm; and (**E**) quantification of aortic iron concentrations (*n* = 5). NC, normal chow; HFCF, hig-fat, high-cholesterol, and high−fructose diet. All data were assessed using Student’s *t*-test and are presented as mean ± SEM. * *p* < 0.05, ** *p* < 0.01, **** *p* < 0.0001.

Prior studies have highlighted iron accumulation in human atherosclerotic plaque areas and its close relationship to atherosclerosis [16]. GO, KEGG, and GSEA analyses revealed dysregulation of the expression of iron absorption and transport genes in the abdominal aorta of the HFCF group of pigs. Notably, we observed a significant increase in the expression of transferrin receptor (TFRC), which is primarily responsible for iron uptake, and iron transporter (SLC40A1), which is primarily responsible for iron export, in the abdominal aorta of the HFCF group. Other genes involved in iron homeostasis, such as ferritin light chain (FTL) and nuclear receptor coactivator 4 (NCOA4), were also significantly upregulated following the HFCF diet (Figure 6A). We corroborated the RNA-Seq results via qRT-PCR, demonstrating that TFRC (*p* < 0.0001), SLC40A1 (*p* < 0.0001), NCOA4 (*p* < 0.01), and FTL (*p* < 0.01) were upregulated in the HFCF group (Figure 6B). Immunohistochemistry results indicated that TFRC and FTL protein expression significantly increased (Figure 6C). Moreover, a significantly higher iron content was found in the abdominal aorta of the HFCF group compared with the NC group, corroborated by Perls’ iron staining (Figure 6D, E). Taken together, these results suggest that HFCF diet feeding leads to iron overload due to the dysregulation of iron metabolism in the abdominal aorta of pigs.

### 3.5. C16 Cer Increases ROS Production, Apoptosis, and Inflammatory Pathway Activation in Macrophages by Inducing Iron Overload

**Figure 7:**
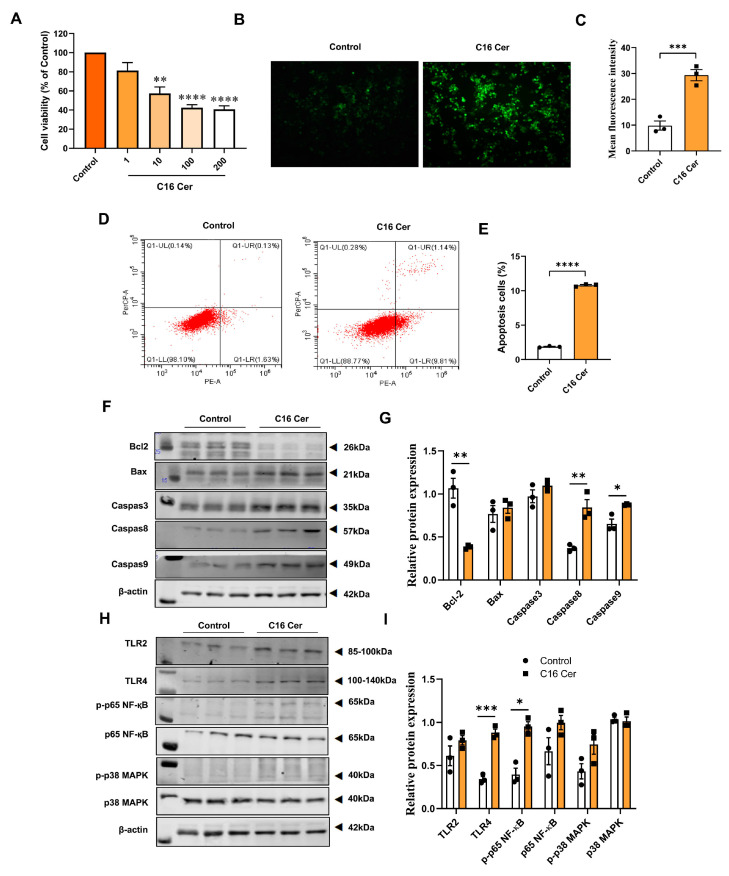
C16 ceramide induces oxidative stress, apoptosis, and inflammatory response in macrophages. (**A**) Cell viability of RAW264.7 cells treated with 1–200 μM of C16 Cer for 24 h (*n* = 6); (**B**) ROS generation with C16 Cer treatment was analyzed via 2′,7′-dichlorodihydro-fluorescein diacetate (DCFH-DA) staining; (**C**) the quantification of ROS levels in RAW264.7 cells (*n* = 3); (**D**) the apoptosis of RAW264.7 cells after C16 Cer treatment was assessed via flow cytometric analysis; (**E**) the relative levels of apoptosis in RAW264.7 cells (*n* = 3); (**F**) immunoblot analysis of Bcl2, Bax, Caspase-3, Caspase-8, and Caspase-9 in RAW264.7 cells; (**G**) the normalized band intensities of Bcl2, Bax, Caspase-3, Caspase-8, and Caspase-9 compared with β-actin (*n* = 3); (**H**) immunoblot analysis of TLR2, TLR4, p-p65 NF-қB, p65 NF-қB, p-p38 MAPK, and p38 MAPK; (**I**) the normalized band intensities of TLR2, TLR4, p-p65 NF-қB, p65 NF-қB, p-p38 MAPK, and p38 MAPK compared with β-actin (*n* = 3). C16 Cer, C16 ceramide. All data were assessed using Student’s *t*-test and are presented as mean ± SEM. * *p* < 0.05, ** *p* < 0.01, *** *p* < 0.001, **** *p* < 0.0001.

While ceramide has been shown to induce apoptosis and inflammation [17], little is currently known about whether it can induce these biological processes by affecting iron deposition, particularly in macrophages. By analyzing human and mouse aortic single-cell RNA-Seq data, we observed that key genes related to ceramide generation and iron deposition were highly expressed in macrophages (Supplementary Materials, S1: Figure S4). Therefore, we investigated whether ceramide affects macrophage iron metabolism, subsequently impacting oxidative stress, apoptosis, and inflammation. RAW264.7 cells were treated with C16 Cer at concentrations ranging from 0 to 200 μM for 24 h (Figure 7A), and 100 μM C16 Cer was selected for the subsequent experiments. DCFH-DA probe results and flow cytometry revealed increased levels of ROS (*p* < 0.001) and apoptosis (*p* < 0.0001) in RAW264.7 cells after C16 Cer treatment (Figure 7B–E). Furthermore, C16 Cer treatment downregulated the protein expression of BCL-2 in RAW264.7 cells (*p* < 0.01). Notably, it significantly upregulated the protein expression levels of Caspase-8 (*p* < 0.01) and Caspase-9 (*p* < 0.05). Additionally, protein expression levels of Bax and Caspase-3 increased after C16 Cer treatment, although the difference was not statistically significant. Moreover, after the C16 Cer treatment, there was a significant increase in the protein expression levels of TLR4 (*p* < 0.001) and p-p65 NF-қ B (*p* < 0.05) in RAW264.7 cells (Figure 7F–I).

**Figure 8:**
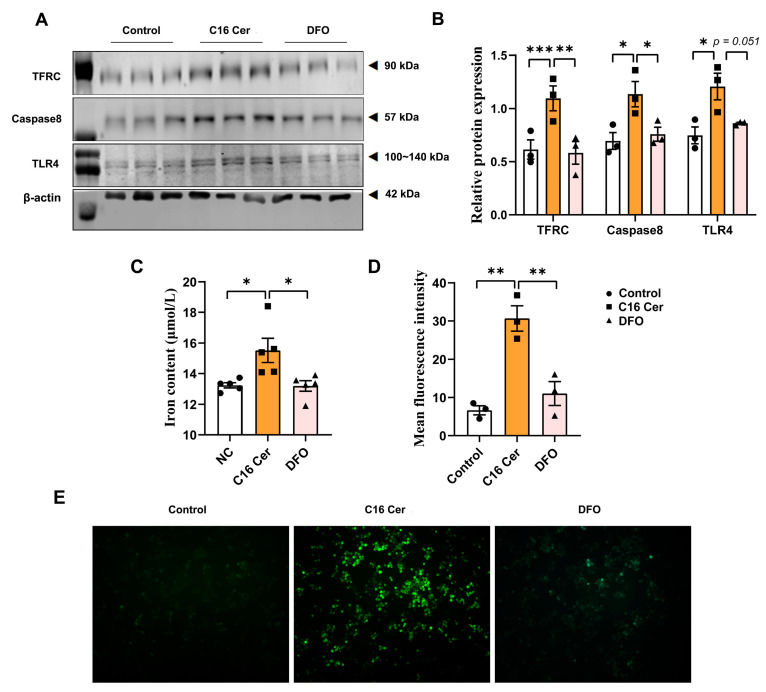
Deferoxamine reverses C16 Cer-induced oxidative stress, apoptosis, and inflammatory response in macrophages. (**A**) Immunoblot analysis of TFRC, Caspase-8, and TLR4; (**B**) the normalized band intensities of TFRC, Caspase-8 and TLR4 compared with β-actin (*n* = 3); (**C**) quantification of iron concentrations in RAW264.7 cells (*n* = 5); (**D**) the quantification of ROS levels in RAW264.7 cells (*n* = 3); and (**E**) ROS generation with C16 Cer and DFO treatment was analyzed via DCFH-DA staining. C16 Cer, C16 ceramide; DFO, deferoxamine. Data are expressed as mean ± SEM. One-way ANOVA analysis was performed followed by Tukey’s post hoc test. * *p* < 0.05, ** *p* < 0.01, *** *p* < 0.001.

To confirm the role of iron in C16 ceramide-induced oxidative stress, apoptosis, and inflammation in macrophages, we pretreated RAW264.7 cells with 50 μM of DFO. The results showed that the C16 Cer treatment significantly increased intracellular iron content (*p* < 0.05) and upregulated the protein expression levels of TFRC (*p* < 0.001) in RAW264.7 cells (Figure 8A–C). Pretreatment with DFO mitigated the increase in TFRC (*p* < 0.01), intracellular iron content (*p* < 0.05), and ROS levels (*p* < 0.01) induced by C16 Cer treatment (Figure 8A–E). Additionally, DFO pretreatment ameliorated the increased protein expression levels of TLR4 (*p* = 0.051) and Caspase-8 (*p* < 0.05) induced by C16 Cer treatment (Figure 8A,B). 

## 4. Discussion

Over the years, pigs have been widely utilized in cardiovascular disease research to study pathological mechanisms and therapeutic drug development for conditions like atherosclerosis. However, there is a dearth of studies investigating the characteristics and biological implications of lipid composition in atherosclerotic plaques in pigs. In this study, we employed LC-MS/MS-based lipidomics and RNA-Seq to expand our understanding of how lipid metabolism is altered in the abdominal aorta tissue of atherosclerotic pigs induced by an HFCF diet. Our results uncovered significant changes in lipid species composition in the aortas of atherosclerotic pigs, particularly sphingolipids, influenced by the length of acyl chains associated with sphingolipids as affected by the HFCF diet. Furthermore, the HFCF diet altered the aortic transcriptional profile, with DEGs enriched in pathways associated with sphingolipid metabolism, cellular iron ion homeostasis, apoptosis, and inflammatory responses. Interestingly, our findings suggested that C16 ceramide could be a key player in promoting iron deposition in macrophages, subsequently triggering increased intracellular ROS production, apoptosis, and the activation of the TLR4/NF-қB inflammatory pathway. These effects may play a crucial role in the pathogenesis of atherosclerosis in pigs induced by the HFCF diet. 

### 4.1. Lipidomics Profiling of Atherosclerotic Plaques from Pigs and Other Mammals

It is widely acknowledged that oxidized low-density lipoprotein (ox-LDL), resulting from the oxidative modification of plasma-derived LDL, plays a significant role in the development of atherosclerosis. In addition to LDL, human atherosclerotic plaques have been found to contain various other lipid types, including sphingolipids and glycerophosphates. In our study, untargeted lipidomics revealed selective remodeling of several lipid classes in the abdominal aorta tissue of pigs subjected to an HFCF diet, including Cer, CerG1, CerG3, AcCa, TG, and LPC. Notably, previous studies have reported variations in the types and quantities of lipid species detected due to differences in detection conditions and sample sources. For example, Stegemann et al. [18] identified 150 lipid species across nine different classes in human carotid endarterectomy specimens using a chip-based robotic nanoelectrospray platform interfaced with a triple quadrupole mass spectrometer (QqQ-MS). In their study, radial arteries were used as control samples, and the identified lipids included cholesterol ester (CE), SM, LPC, and PC. In another study, Edsfeldt et al. [17] utilized high-performance liquid chromatography coupled with tandem mass spectrometry (HPLC-MS/MS) to analyze sphingolipids in human carotid plaques. They found significant increases in Cer, dihydroceramide (dhCer), glucosylceramide (GlcCer), lactosylceramide (LacCer), SM, and sphingosine-1-phosphate (S1P) in symptomatic plaques compared with asymptomatic ones. Additionally, Jung et al. [19] employed ultra-performance liquid chromatography–quadrupole time-of-flight mass spectrometry (UPLC-QTOF-MS) to characterize the global metabolic profile of human aortic tissue samples containing plaques. Their findings showed elevated levels of sphingolipids (Cer, SM, and glucosylceramide) and glycerophospholipids (LPC, LPE, and PC) in aortas with plaques compared with control samples without plaques. Numerous lipidomics studies have been conducted in mouse models of atherosclerosis, with many focusing on peripheral blood samples [20,21,22]. Notably, a recent study utilized LC-MS/MS to assess alterations in the lipid profile of thoracic aorta samples from APOE^−/−^ mice. A total of 131 differential lipids were identified in the samples, including TG (*n* = 53), PE (*n* = 24), PC (*n* = 10), PS (*n* = 10), and SM (*n* = 7) [23]. They found that the levels of all lipids in the PC and LPC classes were significantly increased in the high-fat diet group. However, the results of this study did not align with previous findings in APOE^−/−^ mice, with some discrepancies observed, such as no significant changes in Cer levels. In contrast, Kobayashi et al. [24] discovered higher levels of Cer classes in the aortas of APOE^−/−^ mice compared with wild-type mice, including C16:0 Cer, C18:0 Cer, and C24:0 Cer. It has been reported that there is a significant accumulation of ceramides d18:0 and d18:1 in the aorta of PCSK9^−/−^ mice fed a chow diet. When fed a Western diet, the accumulated ceramides in the aorta were higher in LDLR^−/−^ mice than those in PCSK9^−/−^ mice [25]. Cao et al. [26] identified 33 plaque-specific lipids in high-fat diet-fed LDLR^−/−^ mice with mass spectrometry imaging, including one SM, three LPAs, four LPCs, two LPEs, and one LPI, and found that LPI 18:0 was predominantly localized in the necrotic core of the plaque. Similarly, Martin-Lorenzo et al. [27] also found increased lysoPI (20:3) and SM (d18:0/15:0) in the aortic region of high-fat diet-fed LDLR^−/−^ mice with mass spectrometry imaging. Further studies will need to be performed to investigate the lipid profile in the aorta of patients or atherosclerotic animal models to provide an in-depth understanding of the complex pathogenesis of atherosclerosis.

### 4.2. Changes in PC and LPC in Pig Atherosclerotic Plaques

In the present study, we observed significantly elevated levels of seven LPCs in pigs subjected to the HFCF diet compared with the NC group, including LPC (16:0e), LPC (16:1), LPC (18:0), LPC (18:0e), LPC (18:1), and LPC (18:2). In cells, LPC and PC can be interconverted, with PC being cleaved by phospholipase A2 (PLA2) to form LPC, while LPC can be converted back to PC by lysophosphatidylcholine acyltransferase (LPCAT). Furthermore, we noted alterations in various PC species, with increased PC (16:0/18:1) and PC (16:0/18:2), and decreased PC (18:0p/18:1) and PC (18:0/20:4). Similar results have been reported in studies on diseased iliac arteries from pigs with induced diabetes and hypercholesterolemia, indicating increased arterial LPC content and changes in PC composition, such as LPC (16:0), LPC (18:0), LPC (18:1), LPC (18:2), PC (16:0/18:2), and PC (18:1/18:2) [28]. Additionally, our transcriptome data revealed significantly higher mRNA expression levels of lysophosphatidylcholine acyltransferase 2 (*LPCAT2*), phospholipase A2 group IVA (*PLA2G4A*), and group VII (*PLA2G7*) in the HFCF group of pigs compared with the NC group, consistent with the study by De Keyzer et al. [29], who observed higher expression of PLA2G7 in monocytes and plaque macrophages of miniature pigs fed a cholesterol-rich diet when compared with controls. 

### 4.3. Changes in Gene Expression Related to Sphingolipid Metabolism in Pig Atherosclerotic Plaques

It is well established that the ceramide synthesis pathway is complex, involving multiple processes. It can be generated from plasma membrane sphingomyelin by activating sphingomyelinases or synthesized de novo. Additionally, ceramide can be converted into sphingomyelin by sphingomyelin synthase or further metabolized through ceramidases. In our study, transcriptomic analysis revealed increased expression of genes responsible for ceramide biosynthesis (*CERS6*, *SGPP1*, and *GALC*) and utilization (*ASAH1* and *SGMS1*). Research has shown that mammalian ceramide synthases play a role in the de novo generation of ceramides with specific fatty acid chain lengths. CERS6, in particular, is responsible for the preferential generation of C16 ceramide and has been implicated in atherosclerosis regulation [30]. In our analysis of the aorta transcriptome of APOE^−/−^ mice induced by a Western diet, we found an upregulation of mRNA expression of *CERS6*, consistent with the increase in C16 Cer in the aortas of HFCF-fed pigs. We also observed an upregulation of *SGPP1*, which has been reported to catalyze the degradation of sphingosine-1-phosphate (S1P) through salvage and recycling of sphingosine into long-chain ceramides, having a pro-apoptotic effect by increasing ceramide expression [31]. Additionally, we noted increased expression of ASAH1 and GALC, two lysosomal enzymes—the former cleaving ceramide into sphingosine and free fatty acid, and the latter responsible for degrading galactosylceramide and sphingolipids to produce ceramide [32,33]. SGMS1, a member of the sphingomyelin synthase family, is involved in ceramide metabolism [34]. Previous studies have indicated elevated expression of *ASAH1* in human atherosclerotic plaques [32]. Furthermore, *CERS6*, *ASAH1*, *SGPP1*, and *SGMS1* are significantly enriched in M1-type macrophages within atherosclerotic plaques [35]. These findings suggest a disruption in the balance of ceramide synthesis and degradation in atherosclerotic pig plaques induced by the HFCF diet, potentially leading to transformations between different sphingolipids. Edsfeldt et al. [17] consistently sought to detect the gene expression profile of human atherosclerotic plaque but found no changes in the expression of genes related to sphingolipid metabolism. They speculated that this might be due to the activity of enzymes, especially considering that serum lipid levels in atherosclerosis patients were not significantly increased. 

### 4.4. Dysregulation of Ceramide Metabolism Is Linked to Iron Deposition and Activation of Related Pathways in Macrophages

Ceramide reportedly induces apoptosis and proinflammatory responses in various cell types, particularly in murine and human macrophages, which are significant sites for iron storage. An overload of intracellular iron ions leads to the generation of ROS, resulting in apoptosis and inflammatory responses. In our study, we observed the induction of genes related to iron storage in atherosclerotic pig plaques induced by the HFCF diet, including *TFRC*, SLC40A1, and *FTL*. Treatment with long-chain C16 Cer induced TFRC protein expression and iron deposition in RAW264.7 cells. Moreover, it led to ROS production, apoptosis, and the expression of proteins associated with the TLR4/NF-қB pathway in these cells. Importantly, these changes could be reversed by pretreatment with deferoxamine. Our findings suggest a unique relationship between ceramide and iron in mediating cytotoxicity. In human HepG2 cells, ceramide accumulation promoted hepcidin expression, subsequently increasing intracellular iron content and triggering ceramide production [36]. A previous study by Matsunage et al. [37] demonstrated that C2 Ceramide induced oxidative damage and apoptosis in bovine aortic endothelial cells (BAECs), which DFO could reverse. These findings are in agreement with our results, indicating that ceramide may promote the development of atherosclerosis induced by the HFCF diet by affecting the iron ion balance, subsequently inducing macrophage apoptosis and activating inflammatory pathways. Targeted regulation of ceramide and iron metabolism in macrophages may hold potential clinical value for treating atherosclerosis induced by a high-fructose diet. 

### 4.5. Therapeutic Potential of Ceramide

Ceramides are implicated in a variety of pathological processes associated with cardiovascular disease, including inflammation, oxidative stress, and apoptosis. Studies have shown that the administration of ceramide analogs elevates ROS production in endothelial cells, perhaps owing to NADPH oxidase activation [38]. Studies have also confirmed that ceramide elicited an inflammation response in macrophages [39]. In addition, the findings from studies of animal models suggest that administration of the serine palmitoyltransferase inhibitor myriocin decreased atherosclerotic lesion size in the aorta of APOE^−/−^ mice [40,41]. All these observations support a potential therapeutic role for ceramides in the treatment of atherosclerosis. However, ceramides are indispensable for a variety of biological processes, especially in the nervous and immune systems. Global inhibition of ceramide production is likely not the most feasible approach for treating atherosclerotic cardiovascular disease [42]. In this study, we found that the enzymes responsible for ceramide synthesis were induced in the aorta of atherosclerotic pigs, leading to excessive accumulation of ceramide. Thus, targeting local ceramide production in the vascular tissue to reduce iron deposition and related processes may be more effective. 

### 4.6. Limitations of this Study

Our study has certain limitations. First, we solely verified the expression levels of sphingolipid metabolism genes obtained through RNA sequencing and did not measure their protein or enzyme activity due to a lack of suitable antibodies and reagents for pigs. Second, practical challenges in pig experiments prevented us from directly confirming the relationship between ceramide, apoptosis, and the activation of the TLR4/NF-қB inflammatory pathway in vivo. However, additional in vitro experiments provided compelling evidence that long-chain ceramide plays a crucial role in inducing cellular apoptosis, oxidative stress, and inflammation. Lastly, while we used LC-MS/MS technology to detect nearly 800 lipid species in atherosclerotic pig plaques, some lipid species, including CE, FA, and sphingosine, were identified in small abundance, suggesting that new lipidomics detection technology may be required for further analysis in the future. 

## 5. Conclusions

**Figure 9:**
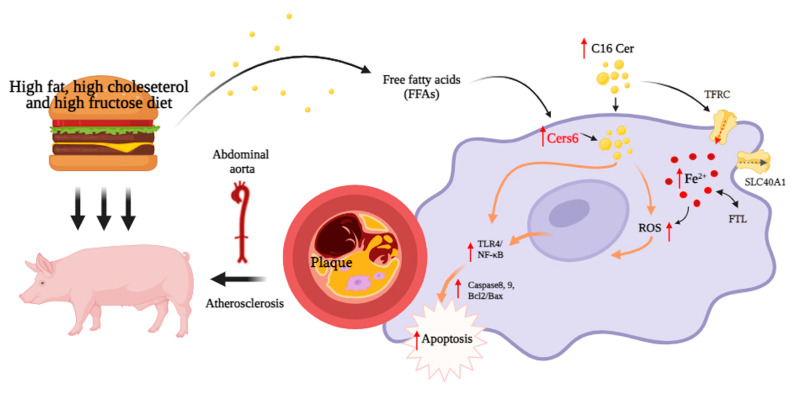
Schematic diagram of the proposed pathological mechanism by which C16 Cer increases ROS production, apoptosis, and inflammation response in macrophages and atherosclerosis by inducing iron overload. C16 Cer, C16 ceramide; CERS6, ceramide synthase 6; TFRC, transferrin receptor; FTL: ferritin light chain; SLC40A1, iron transporter; TLR4, toll-like receptor 4; NF-қB, nuclear factor-kappa B; and ROS, reactive oxygen species.

Overall, our lipidomics data unveiled alterations in the lipid composition of abdominal aorta tissues in HFCF diet-induced atherosclerotic pigs. We identified abnormal ceramide accumulation in atherosclerotic pig plaques, particularly long-chain C16 Cer. Subsequent analysis showed that C16 Cer induces the production of reactive oxygen species in an iron-dependent manner, leading to apoptosis and the activation of the TLR4/NF-қB pathway in macrophages (Figure 9). Transcriptome results confirmed significant changes in gene expression related to sphingolipid metabolism, iron ion balance, apoptosis, and the TLR4/NF-қB pathway. Therefore, targeting C16 ceramide and iron metabolism in macrophages may represent a novel and effective therapeutic approach against atherosclerosis.

## Data Availability

The data supporting the findings of this study are included in the article and supplemental material. Raw RNA-Seq data are available in the Gene Expression Omnibus database (GSE245530).

## Supplementary Materials

The following supporting information can be downloaded at: https://www.mdpi.com/article/10.3390/antiox13010004/s1, Supplementary file S1: Table S1. Information on primers used to perform qRT-PCR; Table S2. The abbreviations of detected lipid classes; Figure S1. Multivariate data analysis of LC-MS/MS lipidomics data from abdominal aorta samples from NC and HFCF pigs; Figure S2. Altered fatty acid composition of sphingolipids in the aorta of miniature pigs fed a HFCF diet; Figure S3. Gene set enrichment analysis showing significant enrichment in iron uptake and transport, toll-like receptor cascades, and apoptosis from NC and HFCF pigs; Figure S4. Ceramide generation and iron deposition related genes are found in macrophage cells in both human and mouse aortas. Supplementary file S2: Table S3. The detected lipid classes; Table S4. Lists of differentially expressed genes (DEGs) between NC and HFCF groups of pigs; Table S5. KEGG pathway analysis of differentially expressed genes (DEGs) between NC and HFCF groups of pigs; Table S6. GO enrichment analysis of differentially expressed genes (DEGs) between NC and HFCF groups of pigs.
