# Supplementary material for: Dysregulation of Ceramide Metabolism Is Linked to Iron Deposition and Activation of Related Pathways in the Aorta of Atherosclerotic Miniature Pigs
Source: Antioxidants (Basel). 2023 Dec 19;13(1):4. doi: 10.3390/antiox13010004 (PMC10812416; doi:10.3390/antiox13010004)
Supplement: Supplementary file 1 [file antioxidants-13-00004-s001.zip › Supplementary file 1.pdf]

Table S1. Information on primers used to perform qRT-PCR

No	Gene symbol	Primer sequences (5'-3')		GenBank No.
1	FTL	Forward	AGAAGATGGGCGACCACCTGAC	NM_001244131.1
		Reversed	TCGTGCTTGAGTGTGAGCCTTTC	
2	NCOA4	Forward	TTGGAGCTTGCTATTGGTGGAGTTC	NM_001243122.1
		Reversed	CTGTGAATCTGGGCTTTGACCTCTC	
3	SLC40A1	Forward	TGTCCCTGAGATGAGCCCTAAACC	XM_003483701.4
		Reversed	AAGACCGATTCTAGCAGCAATGACG	
4	TFRC	Forward	GCTCGGCAGGTAGATGGTGATAAC	NM_214001.1
		Reversed	TGTGGTTACTCCTTGTGTTGCTGTC	
5	ASAH1	Forward	GCTGTCACTGGCATACCTTTAGGAG	XM_003134187.6
		Reversed	TCCACCCAAGCAATAGTCCGAAATC	
6	CERS6	Forward	GACCACAAATTGCCCAGCCAAATG	XM_021075470.1
		Reversed	TCTGTCGGAACCAGTGTTGAATGC	
7	GALC	Forward	TCTGCTTCTATGCTGCTTGACTCTG	NM_001243631.1
		Reversed	ACTGTACGGGTCCCAGGATAATGAG	
8	SGMS1	Forward	GTTGGCACGCTGTACCTGTATCG	NM_001097438.2
		Reversed	ATTATTCTCCGCAGTTGGGCTTCC	
9	SGPP1	Forward	CGTGGTCAAGTTGGAGGTCTTCTAC	XM_013993487.2
		Reversed	CAACGGCCATAGGTGAGCAGAAC	
10	GAPDH	Forward	TCACCAACACCAGCATCCA	NM_001206359.1
12		Reversed	GCACATCTCTCGAAGCACCA	

Note: FTL: Ferritin light chain; NCOA4: Nuclear receptor coactivator 4; SLC40A1: Iron transporter; TFRC: Transferrin receptor; ASAH1: Neutral ceramidase 1; CERS6: Ceramide synthase 6; GALC: galactosylceramidase; SGMS1: Sphingomyelin synthase 1; SGPP1: Sphingosine-1-phosphate phosphatase 1; GAPDH: Glyceraldehyde-3-phosphate dehydrogenase.

Table S2. Abbreviations for the detected lipids

Lipid Class	Lipid Name
PC	Phosphatidylcholine
TG	Triglyceride
PE	Phosphatidylethanolamine
SM	Sphingomyelin
Cer	Ceramides
PS	Phosphatidylserine
PI	Phosphatidylinositol
LPC	Lysophosphatidylcholine
CerG1	hexosyl ceramide
DG	Diglyceride
LPE	Lysophosphatidylethanolamine
PG	Phosphatidylglycerol
CerG3	hexosyl ceramide
CerG3GNAc1	Dihexosyl N-acetylhexosyl ceramide
LPS	Lysophosphatidylserine
GM3	Ganglioside
So	Sphingosine
Co	Coenzyme Q
LPI	Lysophosphatidylinositol
FA	Fatty acid
MGDG	Monogalactosyldiacylglycerol
ChE	Cholesterol Ester
CL	Cardiolipin
AcCa	Acyl Carnitine

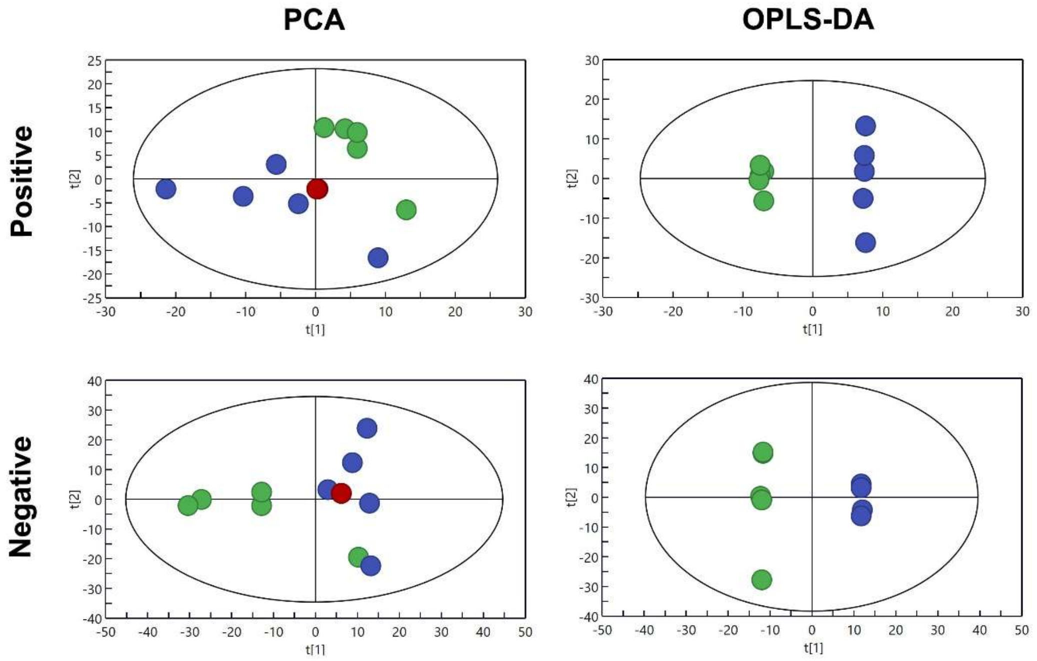

Figure S1: Multivariate data analysis of LC-MS lipidomics data from abdominal aorta samples from NC and HFCF pigs. Unsupervised principal component analysis (PCA) score plot and supervised orthogonal partial least-squares-discriminant analysis (OPLS-DA) models. Blue and green symbols represent aorta samples from HFCF and NC pigs, respectively. Red symbols represent QC samples. NC, normal chow; HFCF, high-fat, high-cholesterol, and high-fructose diet.

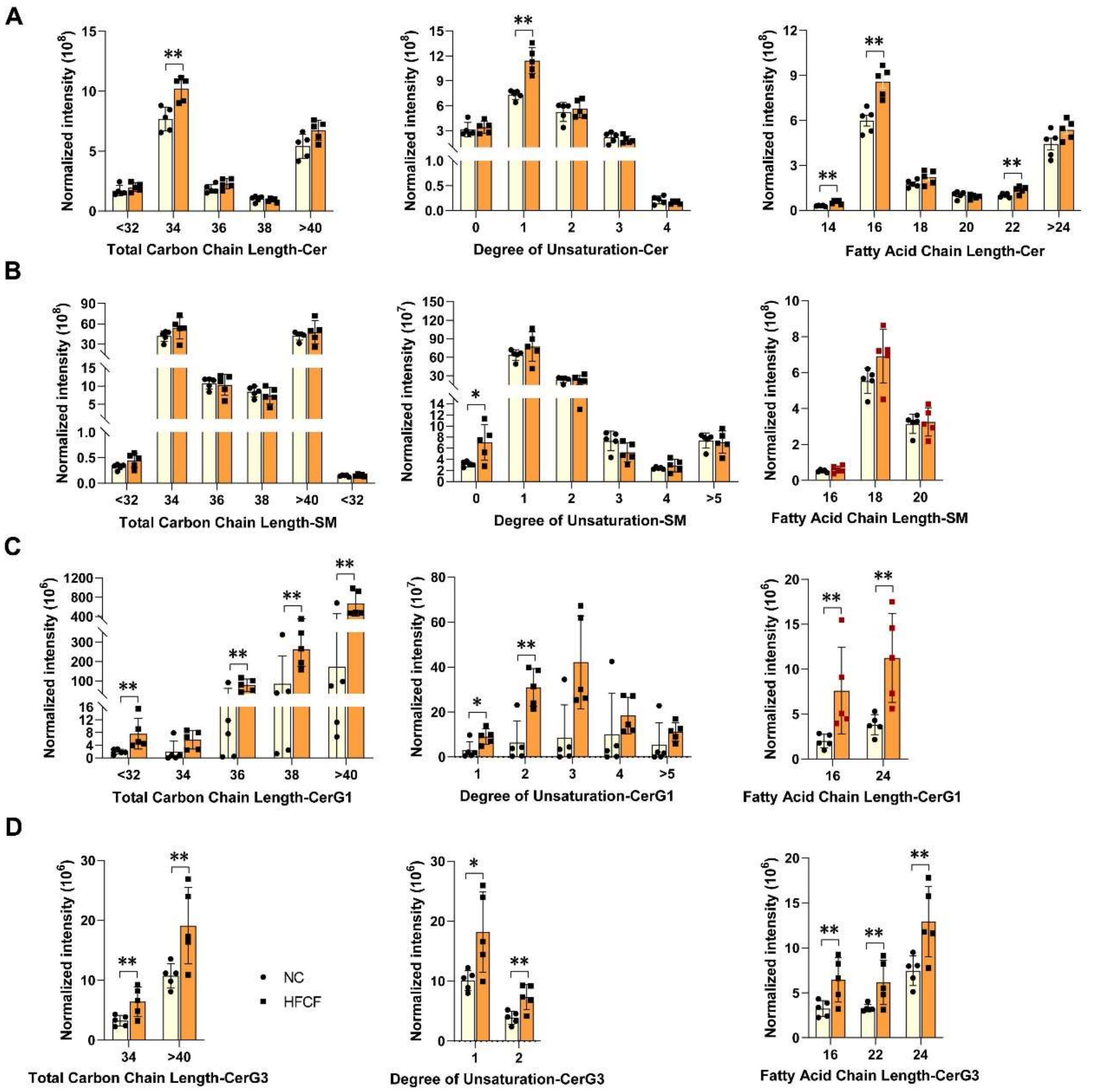

Figure S2: Altered fatty acid composition of sphingolipids in the aorta of miniature pigs fed a HFCF diet. Analysis of fatty-acyl composition of Cer, SM, CerG1, and CerG3 species by total carbon chain length total degree of unsaturation and sphingolipids N-acyl chain lengths (A-D). Data from five pigs for each group were used for statistical analysis. NC, normal chow; HFCF, high-fat, high-cholesterol, and high-fructose diet; Cer, Ceramide; SM, Sphingomyelin; CerG1 and CerG3, hexosyl ceramide. All data were presented as means ± SE and analyzed using student's, * p <0.05, ** p <0.01.

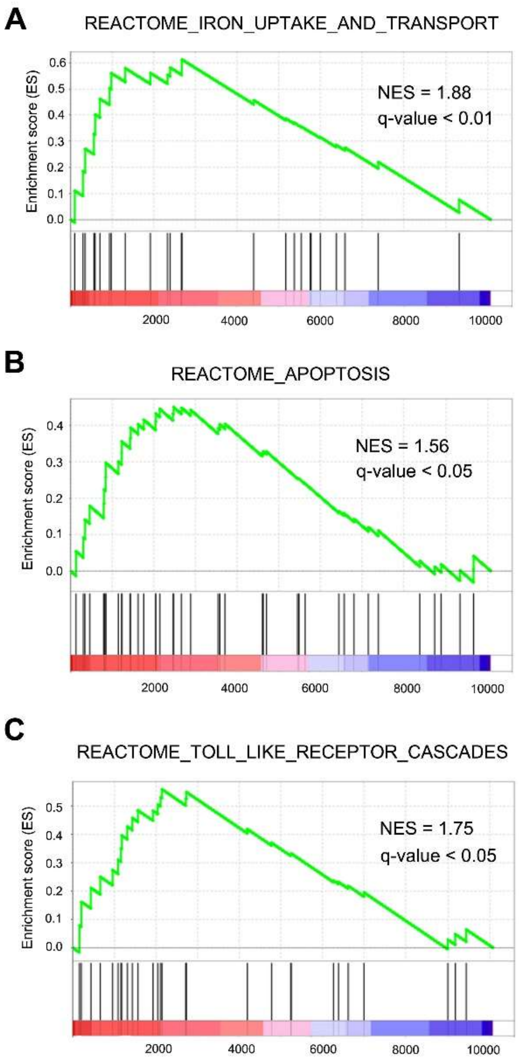

Figure S3: Gene set enrichment analysis showing significant enrichment in iron uptake and transport, toll-like receptor cascades, and apoptosis from NC and HFCF pigs. NC, normal chow; HFCF, high-fat, high-cholesterol, and high-fructose diet. NES, normalized enrichment score.

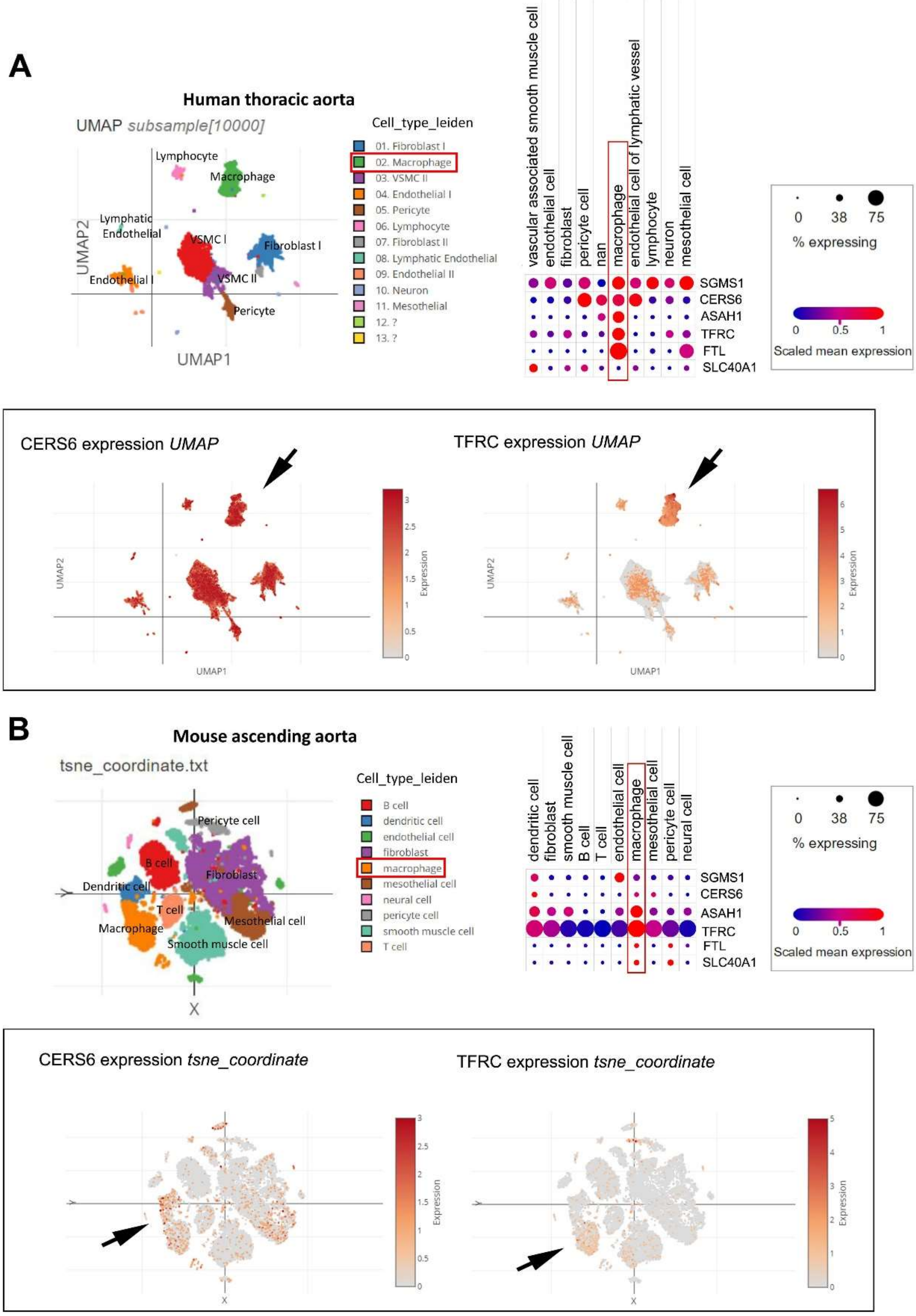

Figure S4: Ceramide generation and iron deposition related genes are found in macrophage cells in both human and mouse aortas. Single-cell RNA-Seq analyses were performed on the Single Cell RNA-Seq database of the Broad Insitute of MIT and Harvard. A. Single-Cell RNA-Seq database portal for human thoracic aorta: https://singlecell.broadinstitute.org/single_cell/study/SCP1265/deep-learning-enables-genetic-

analysis-of-the-human-thoracic-aorta (https://www.biorxiv.org/content/10.1101/2020.05.12.091934v1); B. mouse ascending aorta: https://singlecell.broadinstitute.org/single_cell/study/SCP1361/single-cell-transcriptome-analysis-reveals-cellular-heterogeneity-in-the-ascending-aorta-of-normal-and-high-fat-diet-mice#study-visualize. CERS6, ceramide synthase 6; TFRC, transferrin receptor.
